# Longitudinal Association of Nut Consumption and the Risk of Cardiovascular Events: A Prospective Cohort Study in the Eastern Mediterranean Region

**DOI:** 10.3389/fnut.2020.610467

**Published:** 2021-01-21

**Authors:** Noushin Mohammadifard, Niloufar Ghaderian, Razieh Hassannejad, Firouzeh Sajjadi, Masoumeh Sadeghi, Hamidreza Roohafza, Jordi Salas-Salvadó, Nizal Sarrafzadegan

**Affiliations:** ^1^Isfahan Cardiovascular Research Center, Cardiovascular Research Institute, Isfahan University of Medical Sciences, Isfahan, Iran; ^2^Interventional Cardiology Research Center, Cardiovascular Research Institute, Isfahan University of Medical Sciences, Isfahan, Iran; ^3^Hypertension Research Center, Cardiovascular Research Institute, Isfahan University of Medical Sciences, Isfahan, Iran; ^4^Heart Failure Research Center, Cardiovascular Research Institute, Isfahan University of Medical Sciences, Isfahan, Iran; ^5^Cardiac Rehabilitation Research Center, Cardiovascular Research Institute, Isfahan University of Medical Sciences, Isfahan, Iran; ^6^Department of Biochemistry and Biotechnology, Pere Virgili Institute for Health Research, Rovira i Virgili University, Reus, Spain; ^7^CIBER de Fisiopatología de la Obesidad y la Nutrición (CIBEROBN), Instituto de Salud Carlos III, Madrid, Spain; ^8^Faculty of Medicine, School of Population and Public Health, University of British Columbia, Vancouver, BC, Canada

**Keywords:** nut, cardiovascular disease, myocardial infarction, stroke, ischemic heart disease, chronic, mortality

## Abstract

**Background and Aim:** There are few pieces of evidence on the association between nut consumption and the risk of cardiovascular disease (CVD) in the Eastern Mediterranean Region. This study investigated the relationship of nut consumption with the risk of CVD and all-cause mortality in the Iranian population.

**Methods and Results:** This population-based prospective cohort study was carried out in 6,504 randomly selected participants aged ≥35 years in central Iran (2001–2013) in the framework of the Isfahan Cohort Study. Dietary data were collected by a validated 48-item food frequency questionnaire. Subjects or their next of kin were interviewed biannually, looking for the possible occurrence of cardiovascular events and all-cause mortality. During the median follow-up of 135 months and 52,704.3 person-years, we found a total of 751 CVD events. In unadjusted model, participants in the highest quartile of nut intake had a lower CVD risk {hazard ratio (HR) [95% confidence interval (CI)]: 0.57(0.47–0.70); *P* for trend < 0.001}, CVD mortality [HR (95% CI): 0.54 (0.33–0.72); *P* for trend < 0.001], and all-cause mortality [HR (95% CI): 0.24 (0.14–0.42); *P* for trend < 0.001]. In the fully adjusted model, the association was diluted, and no significant relationship was found between nut intake and CVD events and all-cause mortality, except for CVD mortality in the highest quartile vs. the lowest one [HR (95% CI): 0.55 (0.30–0.98)].

**Conclusion:** Nut intake had an inverse association with the risk of CVD mortality. It is suggested to perform studies to examine the association of individual types of nuts and different preparation methods on CVD risk and mortality.

## Introduction

Globally, cardiovascular diseases (CVDs) are the leading cause of death and one of the major health concerns (1, 2). It is well-established that dietary factors are of great importance in developing, preventing, and managing CVD (3). Healthy dietary patterns are associated with lowered cardiovascular morbidity and mortality (4), and fats are one of the dietary factors that have been the focus of several studies. Replacing saturated fat with polyunsaturated fatty acid (PUFA) and monounsaturated fatty acid (MUFA) has been consistently reported to lower the incidence of CVD (5).

Nuts are a good source of unsaturated fatty acids with low amounts of saturated fats. They also contain many other components that may reduce CVD risk (6). These compounds include protein, minerals (calcium, magnesium, and potassium), vitamins (folic acid, niacin, tocopherols, and vitamin B-6), fiber, phytosterols, and polyphenols (7). The presence of nuts in the diet may decrease the CVD mortality rates through possible anti-inflammatory, antioxidant, and antiatherogenic mechanisms (7). Epidemiological studies have consistently shown that nut consumption is associated with a reduced risk of ischemic heart disease (IHD) and CVD incidence and mortality, and also total mortality in developed countries (8).

Several studies indicated the inverse association between nut consumption and CVD risk factor occurrence among the Iranian population (9, 10). However, to our best knowledge, few studies have evaluated the frequency of nut intake and the risk of CVD in Iran and the other countries in the Eastern Mediterranean region. Thus, we investigated whether there is an association between the frequency of nut consumption and the risk of cardiovascular events as well as cardiovascular and all-cause mortality among the Iranian population in this prospective cohort study.

## Subjects and Methods

### Design and Study Population

The study sample consisted of 6,504 adults aged ≥35 years at baseline, living in urban and rural areas in central Iran (Isfahan, Arak, and Najafabad) from January to September 2001 (11). Subjects were chosen using stratified cluster random sampling. Firstly, the population was stratified based on the living area (urban and rural) in each county. The next step was the random selection of censuses from each county. The probability of selection was proportional to the expected number of households and divided into clusters of ~1,000 households. Randomly 5–10% of households in each cluster were selected and counted, and from each household, one eligible individual (Iranian, mentally competent, and not pregnant) was selected randomly.

The home-interviews had a response rate of 98%, but 95% attended the examination clinic. For the present study, the individuals with a previous history of CVD (*n* = 181) and also those who missed before the first follow-up (*n* = 891) were excluded from the study. Eventually, the analysis was conducted on the data collected from 5,432 (84%) participants. At baseline, data were collected through face-to-face interviews, which included information about dietary intake. Afterward, follow-up surveys were carried out every 2 years by telephone interviews. In case of unsuccessful attempts by telephone, home-interviews were done. Also, we carried out repeated measurements similar to the baseline for all participants every 6 years in 2007 and 2013. The study was approved by the Ethics Committee of the Research Council of Isfahan Cardiovascular Research Center, a World Health Organization (WHO) collaborating center in Isfahan, Iran. The study design and details of subjects' recruitment and data collection methods were presented elsewhere (11).

### Dietary Assessment

At baseline, data on dietary intake were collected by a Block-format validated 48-item food frequency questionnaire (FFQ) (12). The FFQs were completed by trained interviewers in a face-to-face manner. The participants, in an open-ended format, reported the frequency of the consumption of the food items during the last preceding year on daily, weekly, or monthly basis. Nut consumption was calculated by questions on walnut, almond, pistachio, and hazelnut consumption. Trained nutritionists rechecked and computed nut intake. The data on portion sizes of food items were not reported, as studies have shown that for most foods, portion size varies less among individuals than do frequencies of consumption. We examined dietary intake at baseline and during repeated measurement in 2007 and 2013 years.

### Ascertainment of Cardiovascular Disease Events

The follow-up of participants continued until the occurrence of CVD events or their last successful interview, mostly before 2013, whichever happened first. The median duration of follow-up was 135 (interquartile range: 93–147) months. Over the 13 years of follow-up, subjects or their families had six biannual telephone interviews. Trained nurses performed structured primary interviews with three main questions: “is he/she alive?” has he/she been hospitalized for any reason? Specifically, focus on cardiovascular and cerebrovascular events. In the occurrence of death or hospitalization, the nurses obtained the date of the events, physician diagnosis, and the hospital's name during the interviews. If the death occurred out of the hospital, death certificates were attained from the provincial mortality database, and a trained expert nurse conducted verbal autopsy including medical history and signs and symptoms before death through a secondary interview with surviving family members. In the case of hospitalization events, trained nurses obtained related documents about reported hospitalization events through medical records and hospital records.

The documents related to each event were evaluated by the specialists' outcome adjudication panel consisted of four cardiologists and two neurologists (11). In the present study, cardiovascular events were defined as fatal and non-fatal myocardial infarction (MI), fatal and non-fatal stroke (which was defined as death from cerebrovascular disease), unstable angina, and sudden cardiac death, using modified criteria of WHO Expert Committee (13). Our definition for MI was based on the presence of at least two of the following criteria: (1) typical chest pain lasting more than 30 min, (2) ST elevation > 0.1 mV in at least two adjacent electrocardiograph leads, and (3) an increase in serum level of cardiac biomarkers. Death within 1 h of onset, a witnessed cardiac arrest, or abrupt collapse not preceded by >1 h of symptoms were considered as sudden cardiac death. Moreover, the WHO stroke definition was used, i.e., stroke was defined as a rapid-onset focal neurological disorder persisting at least 24 h that had a probable vascular origin.

### Assessment of Covariates

Trained health professionals conducted detailed home interviews at the baseline of the study for obtaining the required information about participants' general characteristics (socioeconomic and demographic information, data on dietary behaviors, smoking, and physical activity) (14). Participants were categorized into three groups based on their smoking status: smoker, ex-smoker, and non-smoker. Smokers were smoking at least one cigarette per day at the time of the study; those who previously smoked at least one cigarette per day were defined as ex-smokers and others as non-smokers. A validated physical activity questionnaire was used to assess physical activity (15). Total daily physical activity was calculated in metabolic equivalents of task-minute/day. Medical interviews and physical examinations, including a history of CVD and its risk factors, observations regarding heart rate, blood pressure, and electrocardiography, were obtained by trained physicians and nurses.

Blood pressure was measured according to standard protocols, using a calibrated sphygmomanometer twice after 5 min resting, and the mean was calculated as individual blood pressure. Height was measured without shoes to the nearest 0.5 cm, and weight was measured without shoes and with light clothing with a precision of 0.1 kg using a Seca scale. Body mass index (BMI) was calculated as weight in kilograms divided by square of height in meters (kilograms per square meter). Fasting blood samples were taken and centrifuged immediately in each county; samples obtained in Isfahan and Najafabad were transported to the central laboratory within ~1 h. In Arak, fasting blood glucose and 2-h post-prandial glucose test were measured immediately, and serum was frozen at −20°C, then by a 3-h ground transport with cold chain (−20°C), they were transported to the Isfahan Cardiovascular Research Center central laboratory, which has external national and international quality controls. Serum total cholesterol and fasting blood glucose were measured enzymatically, using an autoanalyzer (Eppendorf, Hamburg, Germany). Those individuals with fasting plasma glucose levels of ≥126 mg/dL or the 2-h post-load plasma glucose ≥200 mg/dl or on insulin or oral hypoglycemic agents were considered as having diabetes mellitus. Having a blood pressure ≥ 140/90 mmHg (based on an average of two measurements) or the use of antihypertensive medications was defined as hypertension. Hypercholesterolemia was defined as having serum total cholesterol levels ≥ 200 mg/dl or statin use.

### Statistical Analysis

Energy-adjusted nut intake was calculated based on BMI as a surrogate measure (16) using the residual method due to the lack of data on energy intake in this study. Individuals were categorized based on the quartiles of nuts-BMI adjusted intake. Quantitative variables were expressed as mean ± SD, and qualitative variables were expressed as a percent. A Chi-square test was applied to compare the distribution of categorical variables across quartiles of nut intake. In terms of continuous variables, we applied the Kruskal–Wallis or Brown–Forsyth tests to evaluate means across quartiles of nut intake when the assumptions of one-way analysis of variance were not met. Analysis of covariance was applied to obtain age- and sex-adjusted intakes of food and food groups across quartiles of nut intake.

Cox regression models were fitted to examine the association between nut intake and cardiovascular events, with time to events as the time variable and the nut intake as the independent variable. The model was stratified on sex—that is, separate Cox regression was applied for men and women. The HRs and 95% CI for cardiovascular events across quartiles of nut intake were estimated based on four modeling processes: (1) crude model; (2) adjusted model by age (continuous) and also sex (male/female) in total population; (3) additionally adjusted by education (illiterate, primary school, and higher than primary school), residence area (urban/rural), smoking status (never, ex-smoker, current smoker), daily physical activity (continuous, metabolic equivalents of task-min/day), family history of CVD (yes/no), diabetes mellitus (yes/no), hypertension (yes/no), hypercholesterolemia (yes/no) and aspirin use (yes/no), and menopausal status in females (yes/no); (4) further adjustment for BMI and dietary factors including red meat, fish, fruit and vegetable, hydrogenated and non-hydrogenated vegetable oil, fast food, cereals, legumes, animal fats, sweets, soft drink, and beverages.

The Schoenfeld residuals were used for the testing of the proportional hazards (PH) assumption. Further evaluation was applied to check the PH assumption graphically with the log-cumulative hazard plots against survival time and comparing Kaplan–Meier survival estimates and Cox adjusted estimated plotted on the same graph. If the graphical approaches suggested that there is some violation with PH assumption, extended Cox model was run based on an appropriate function of survival time by defining product term involving time-independent variable with some function of time [g(t) or Heaviside function] and testing the coefficient of the product term. In our analysis, there was not any violation of the PH assumption.

To determine the linear trend of relative risks across quartiles of nut intake, we considered the nut intake as a continuous variable in Cox regression models.

Statistical analyses were performed, using SAS software, version 9.4 (SAS Institute Inc.), and STATA software, version 14. *P* < 0.05 (two-tailed) were considered as statistically significant.

## Results

The baseline characteristics of participants according to the quartiles of nut intake was demonstrated in Table 1. Compared with those in the first quartile of nut intake, individuals in the top quartile had a lower mean of age and BMI and more physically active. They were less likely to be urbanization, illiterate, obese, hypertensive, diabetic, hypercholesterolemic, and postmenopausal women, and more being male and smokers (*P* < 0.001).

**Table 1:** Baseline characteristics of study participants across nuts quartiles.

	**Nut intake**				
	**Q1**	**Q2**	**Q3**	**Q4**	***P*-value**
Age (year)	52.9 ± 11.2	54.47 ± 12.9	48.8 ± 10.8	46.6 ± 9.6	<0.001^a^
Body mass index (kg/m^2^)	30.1 ± 3.4	23.9 ± 3.6	26.30 ± 4.2	26.5 ± 4.2	<0.001^b^
Daily physical activity (METs- min/day)	754.4 ± 480.4	839.3 ± 543.1	937.02 ± 573.8	958.74 ± 568.1	<0.001^a^
Men (%)	35	56	54.1	49.9	<0.001^c^
Urbanization (%)	75.5	65.3	77	71.6	<0.001^c^
Illiterate (%)	46.4	47.3	27.1	24.8	<0.001^c^
Family history of CVD (%)	5.9	4.5	5.1	6	0.26^c^
Current smoker (%)	9.7	19.9	15.9	19.4	<0.001^c^
Obese (BMI ≥ 30) (%)	42.6	6.5	18.8	20	<0.001^c^
Diabetes mellitus (%)	17	9.9	7.8	9.3	<0.001^c^
Hypertension (%)	41.6	29	27	19	<0.001^c^
Hypercholesterolemia (%)	67	56.1	55.6	53.6	<0.001^c^
Post-menopausal women (%)	56.2	56.3	38.9	30.4	<0.001^c^

*Quantitative variables were expressed as Mean ± SD and qualitative variables were expressed as percent*.

a *Obtained from Kruskal-Wallis test*.

b *Obtained from Brown-Forsyth test*.

c *Obtained from Chi-Square test*.

Analyzing the participants' dietary intake indicated that those at the top quartile of nut intake had a higher mean intake of non-hydrogenated vegetable oil, fish, fruits and vegetables, legumes, hydrogenated vegetable oil, red meat, fast foods, animal fats, sweets, soft drinks and beverages (all *P* <0.001), and cereals (*P* = 0.002) (Table 2).

**Table 2:** Adjusted mean of food intake of study participants across nuts intake quartiles.

	**Nut intake**				
	**Q1**	**Q2**	**Q3**	**Q4**	*P*-value^*^
HVO	8.02 ± 0.027	8.16 ± 0.027	8.15 ± 0.027	8.20 ± 0.027	<0.001
NHVO	2.55 ± 0.027	2.38 ± 0.024	2.53 ± 0.027	2.63 ± 0.031	<0.001
Cereals	23.52 ± 0.027	23.58 ± 0.027	23.61 ± 0.027	23.67 ± 0.028	0.002
Red meat	4.03 ± 0.026	4.13 ± 0.028	4.19 ± 0.026	4.30 ± 0.029	<0.001
Fish	0.43 ± 0.029	0.29 ± 0.023	0.43 ± 0.027	0.57 ± 0.032	<0.001
Fruits & vegetables	13.10 ± 0.027	13.03 ± 0.028	13.09 ± 0.025	13.38 ± 0.029	<0.001
Nuts	0.64 ± 0.004	0.66 ± 0.003	0.96 ± 0.004	2.28 ± 0.04	<0.001
Legumes	2.98 ± 0.027	3.07 ± 0.028	3.03 ± 0.025	3.22 ± 0.029	<0.001
Fast foods	0.45 ± 0.028	0.42 ± 0.021	0.54 ± 0.023	0.83 ± 0.033	<0.001
Animal fats	2.42 ± 0.023	2.57 ± 0.028	2.64 ± 0.024	2.89 ± 0.032	<0.001
Sweets, soft drink, & beverages	3.16 ± 0.022	3.21 ± 0.024	3.38 ± 0.024	3.78 ± 0.034	<0.001

*HVO, Hydrogenated vegetable oil; NHVO, Non-hydrogenated vegetable oil*.

*Data are expressed as Mean ± SE; all values are adjusted by age and sex*.

* *Obtained from ANCOVA*.

During the median follow-up of 135 (interquartile range: 93–147) months and 52,704.3 person-years, we found a total number of 751 CVD events. Multivariable-adjusted HRs for MI, IHD, stroke, CVD incidents, all-cause mortality, and CVD mortality across quartiles of nut intake are presented in Table 3. In crude model, participants who ate more nuts had a lower risk of MI [HR (95% CI): 0.50 (0.31–0.81); *P* for trend = 0.002], IHD [HR (95% CI): 0.61 (0.48–0.77); *P* for trend < 0.001], stroke [HR (95% CI): 0.49 (0.31–0.77); *P* for trend < 0.001], CVD [HR (95% CI): 0.57(0.47–0.70); *P* for trend < 0.001], all-cause mortality [HR (95% CI): 0.24 (0.14–0.42); *P* for trend < 0.001], and CVD mortality [HR (95% CI): 0.24 (0.14–0.42); *P* for trend < 0.001] in full adjusted model (Table 3).

**Table 3:** Multiple adjusted hazard ratios of cardiovascular events according to quartiles of nut intake (time per week).

	**Events rate**		**Crude model**	**Model 1^a^**	**Model 2^b^**	**Model 3^c^**
	***N***	**Person-year**	**HR (95% CI)**	**HR (95% CI)**	**HR (95% CI)**	**HR (95% CI)**
**MI**
MI
Q1	50	13711.9	1	1	1	1
Q2	45	13198.9	0.94 (0.63–1.40)	0.74 (0.49–1.12)	0.92 (0.61–1.41)	0.98 (0.61–1.59)
Q3	35	13907.8	0.69 (0.45–1.06)	0.73 (0.47–1.14)	0.89 (0.57–1.40)	0.90 (0.56–1.46)
Q4	26	14198.5	0.50 (0.31–0.81)	0.61 (0.38–1)	0.75 (0.46–1.23)	0.77 (0.45–1.30)
*P* for trend			0.002	0.05	0.28	0.31
PH assumption			0.69	0.81	0.99	0.82
**IHD**
Q1	180	13201.3	1	1	1	1
Q2	166	12807.1	0.95 (0.77–1.18)	0.81 (0.66–1.01)	0.98 (0.78–1.21)	1.07 (0.84–1.37)
Q3	133	13518.9	0.72 (0.57–0.90)	0.83 (0.66–1.04)	0.96 (0.76–1.21)	1.01 (0.79–1.28)
Q4	115	13785.5	0.61 (0.48–0.77)	0.80 (0.63–1.02)	0.93 (0.73–1.19)	0.98 (0.76–1.27)
*P* for trend			<0.001	0.07	0.56	0.81
PH assumption			0.90	0.80	0.65	0.67
**Stroke**
Q1	57	13711.5	1	1	1	1
Q2	44	13187.9	0.81 (0.55–1.20)	0.72 (0.48–1.07)	0.91 (0.60–1.36)	1.15 (0.72–1.82)
Q3	27	13950.8	0.46 (0.29–0.73)	0.59 (0.37–0.93)	0.73 (0.46–1.17)	0.86 (0.53–1.41)
Q4	29	14198.1	0.49 (0.31–0.77)	0.72 (0.45–1.13)	0.95 (0.59–1.51)	1.13 (0.68–1.87)
*P* for trend			<0.001	0.07	0.52	0.96
PH assumption			0.48	0.28	0.35	0.19
**CVD**
Q1	237	13008.6	1	1	1	1
Q2	210	12612.9	0.92 (0.76–1.11)	0.79 (0.65–0.95)	0.97 (0.80–1.17)	1.11 (0.89–1.38)
Q3	160	13425.4	0.65 (0.53–0.79)	0.77 (0.63–0.94)	0.91 (0.74–1.11)	0.98 (0.79–1.22)
Q4	144	13657.4	0.57 (0.47–0.70)	0.78 (0.63–0.96)	0.93 (0.75–1.16)	1.02 (0.81–1.28)
*P* for trend			<0.001	0.013	0.41	0.86
PH assumption			0.84	0.73	0.89	0.79
**All-cause mortality**
Q1	129	13899.8	1	1	1	1
Q2	181	13353	1.47 (1.17–1.84)	1.12 (0.89–1.42)	1.21 (0.95–1.53)	1.05 (0.79–1.39)
Q3	88	14039.5	0.67 (0.51–0.88)	0.89 (0.68–1.18)	0.99 (0.75–1.31)	0.91 (0.67–1.22)
Q4	60	14326.1	0.45 (0.33–0.61)	0.77 (0.56–1.05)	0.84 (0.61–1.16)	0.79 (0.56–1.12)
*P* for trend			<0.001	0.06	0.23	0.12
PH assumption			0.56	0.58	0.60	0.27
**CVD mortality**
Q1	60	13899.8	1	1	1	1
Q2	68	13353	1.19 (0.84–1.68)	0.88 (0.61–1.26)	1.11 (0.77–1.60)	1.04 (0.67–1.60)
Q3	36	14039.5	0.59 (0.39–0.89)	0.77 (0.50–1.17)	0.97 (0.63–1.48)	0.91 (0.58–1.44)
Q4	15	14326.1	0.24 (0.14–0.42)	0.41 (0.23–0.73)	0.54 (0.30–0.97)	0.55 (0.30–0.98)
*P* for trend			<0.001	0.003	0.08	0.08
PH assumption			0.37	0.69	0.48	0.58

*Q, Quartile; HR (95% CI), Hazard ratio (95% confidence interval); MI, Myocardial Infraction; IHD, Ischemic heart disease (MI + Sudden cardiac death+ Unstable angina); CVD, Cardiovascular disease*.

a *Adjusted for age(year) and sex (men/women)*.

b *Additionally, adjusted for education (illiterate/primary school/more than primary school), residency (urban/rural), smoking status (never/past/current smoker), daily physical activity (METs-min/day), family history of cardiovascular disease (yes/no), diabetes mellitus (yes/no), hypertension (yes/no), hypercholesterolemia (yes/no), and aspirin use*.

c *Additionally, adjusted for body mass index (kg/m^2^), dietary factor including red meat, fish, fruit and vegetable, hydrogenated and non-hydrogenated vegetable oil, fast food, cereals, legumes, animal fats, sweets, soft drink, and beverages*.

After adjustment for age and sex, those in highest nut quartiles were significantly less likely to have CVD [HR (95% CI): 0.78 (0.63–0.96); P for trend = 0.013] events and also CVD mortality [HR (95% CI): 0.41 (0.23–0.73); P for trend = 0.003]. In the other two adjusted models, the association was diluted, and no significant relationship was found between nut intake and MI, IHD, stroke, CVD incidents, and all-cause mortality. However, there was a lower risk of CVD mortality in those with the most frequent of nut consumption, compared to the lowest quartile [HR (95% CI): 0.54 (0.30–0.97) in model 2 and 0.55 (0.30–0.98) in full adjustment model], however the trend was not significant (P = 0.08) (Table 3).

Sensitivity analysis based on sex illustrated a significant inverse relationship between frequency of nut intake and risk of MI [HR (95% CI): 0.42 (0.20–0.85); P for trend = 0.004], IHD [HR (95% CI): 0.61 (0.44–0.86); P for trend = 0.002], stroke [HR (95% CI): 0.46 (0.25–0.85); P for trend = 0.003], CVD [HR (95% CI): 0.56 (0.42–0.76); P for trend < 0.001], all-cause mortality [HR (95% CI): 0.48 (0.31–0.76); P for trend < 0.001], and CVD mortality [HR (95% CI): 0.28 (0.11–0.67); P for trend = 0.002] in women. However, these associations disappeared after adjustment for all potential confounders (Table 4). Table 5 shows that, in men, there was a significant relationship between more frequent consumption of nut and lower risk of IHD [HR (95% CI): 0.54 (0.39–0.74); P for trend < 0.001], CVD [HR (95% CI): 0.53 (0.40–0.71); P for trend < 0.001], all-cause mortality [HR (95% CI): 0.37 (0.24–0.56); P for trend < 0.001], and CVD mortality [HR (95% CI): 0.18 (0.09–0.38); P for trend = 0.002]. After full adjustment, we only found a significant inverse association between nut intake and risk of all-cause [HR (95% CI): 0.60 (0.38–0.96); P for trend = 0.05] and CVD mortality [HR (95% CI): 0.37 (0.17–0.83); P for trend = 0.01].

**Table 4:** Multiple adjusted hazard ratios of cardiovascular events according to quartiles of nut intake (time per week) for women.

	**Events rate**		**Crude model**	**Model 1^a^**	**Model 2^b^**	**Model 3^c^**
	**N**	**Person-year**	**HR (95% CI)**	**HR (95% CI)**	**HR (95% CI)**	**HR (95% CI)**
**MI**
Q1	30	8975.1	1	1	1	1
Q2	10	5823.1	0.52 (0.25–1.06)	0.49 (0.24–1.01)	0.55 (0.27–1.15)	0.52 (0.22–1.21)
Q3	8	6393.6	0.37 (0.17–0.82)	0.49 (0.22–1.08)	0.59 (0.27–1.32)	0.56 (0.24–1.30)
Q4	10	7172.9	0.42 (0.20–0.85)	0.62 (0.30–1.30)	0.80 (0.37–1.72)	0.72 (0.31–1.67)
P for trend			0.004	0.08	0.33	0.31
PH assumption			0.61	0.85	0.81	0.55
**IHD**
Q1	100	8655.2	1	1	1	1
Q2	64	5666.7	0.98 (0.72–1.34)	0.95 (0.69–1.30)	1.13 (0.82–1.55)	1.26 (0.87–1.80)
Q3	52	6211.9	0.72 (0.51–1.01)	0.97 (0.69–1.37)	1.10 (0.78–1.55)	1.15 (0.80–1.65)
Q4	50	6948.2	0.61 (0.44–0.86)	0.96 (0.67–1.36)	1.18 (0.83–1.69)	1.22 (0.83–1.79)
P for trend			0.002	0.80	0.36	0.34
PH assumption			0.46	0.37	0.37	0.24
**Stroke**
Q1	38	8940.2	1	1	1	1
Q2	16	5791.4	0.65 (0.36–1.17)	0.64 (0.35–1.14)	0.75 (0.42–1.36)	1.14 (0.59–2.17)
Q3	12	6384.1	0.44 (0.23–0.84)	0.54 (0.28–1.05)	0.67 (0.35–1.31)	0.86 (0.43–1.71)
Q4	14	7147.4	0.46 (0.25–0.85)	0.62 (0.33–1.17)	0.74 (0.38–1.44)	0.95 (0.46–1.93)
P for trend			0.003	0.06	0.24	0.77
PH assumption			0.40	0.37	0.21	0.16
**CVD**
Q1	138	8511.2	1	1	1	1
Q2	80	5609.1	0.88 (0.67–1.16)	0.85 (0.65–1.12)	1.01 (0.77–1.34)	1.23 (0.90–1.69)
Q3	65	6182.2	0.63 (0.47–0.85)	0.84 (0.62–1.13)	0.97 (0.71–1.31)	1.08 (0.78–1.48)
Q4	64	6880	0.56 (0.42–0.76)	0.86 (0.63–1.16)	1.06 (0.78–1.46)	1.15 (0.82–1.62)
P for trend			<0.001	0.24	0.81	0.48
PH assumption			0.76	0.71	0.84	0.67
**All-cause mortality**
Q1	67	9084.2	1	1	1	1
Q2	76	5847.1	1.78 (1.28–2.47)	1.65 (1.19–2.30)	1.73 (1.24–2.41)	1.44 (0.96–2.17)
Q3	28	6409	0.59 (0.38–0.91)	0.95 (0.61–1.48)	1.02 (0.65–1.60)	0.95 (0.58–1.54)
Q4	26	7215.5	0.48 (0.31–0.76)	0.99 (0.62–1.58)	1.17 (0.73–1.87)	1.17 (0.69–1.95)
P for trend			<0.001	0.85	0.61	0.92
PH assumption			0.73	0.86	0.99	0.51
**CVD mortality**
Q1	27	9084.2	1	1	1	1
Q2	27	5847.08	1.57 (0.92–2.67)	1.44 (0.84–2.46)	1.68 (0.98–2.90)	1.59 (0.83–3.02)
Q3	14	6409	0.73 (0.38–1.39)	1.18 (0.61–2.27)	1.45 (0.75–2.83)	1.62 (0.78–3.33)
Q4	6	7215.5	0.28 (0.11–0.67)	0.58 (0.23–1.41)	0.84 (0.33–2.12)	1.03 (0.39–2.72)
P for trend			0.002	0.49	0.71	0.53
PH assumption			0.52	0.76	0.55	0.82

*Q, Quartile; HR (95% CI), Hazard ratio (95% confidence interval); MI, Myocardial Infraction; IHD, Ischemic heart disease (MI + Sudden cardiac death); CVD, Cardiovascular disease*.

a *Adjusted for age(year)*.

b *Additionally, adjusted for education (illiterate/primary school/more than primary school), residency (urban/rural), smoking status (never/past/current smoker), daily physical activity (METs–min/day), family history of cardiovascular disease (yes/no), diabetes mellitus (yes/no), hypertension (yes/no), hypercholesterolemia (yes/no), aspirin use, and post-menopause (yes/no)*.

c *Additionally, adjusted for body mass index (kg/m^2^), dietary factor including red meat, fish, fruit and vegetable, hydrogenated and non–hydrogenated vegetable oil, fast food, cereals, legumes, animal fats, sweets, soft drink, and beverages*.

**Table 5:** Multiple adjusted hazard ratios of cardiovascular events according to quartiles of nut intake (time per week) for men.

	**Events rate**		**Crude model**	**Model 1^a^**	**Model 2^b^**	**Model 3^c^**
	***N***	**Person-year**	**HR (95% CI)**	**HR (95% CI)**	**HR (95% CI)**	**HR (95% CI)**
**MI**						
Q1	20	4736.8	1	1	1	1
Q2	35	7375.8	1.12 (0.65–1.95)	0.99 (0.57–1.74)	1.27 (0.72–2.26)	1.47 (0.76–2.81)
Q3	27	7514.2	0.85 (0.48–1.52)	0.98 (0.55–1.75)	1.19 (0.66–2.16)	1.26 (0.67–2.37)
Q4	16	7025.6	0.54 (0.28–1.04)	0.68 (0.35–1.33)	0.82 (0.42–1.62)	0.88 (0.43–1.81)
*P* for trend			0.03	0.29	0.57	0.60
PH assumption			0.80	0.85	0.79	0.96
**IHD**						
Q1	80	4546.1	1	1	1	1
Q2	102	7140.3	0.81 (0.61–1.09)	0.72 (0.53–0.96)	0.88 (0.65–1.20)	0.95 (0.67–1.35)
Q3	81	7307	0.63 (0.46–0.85)	0.72 (0.53–0.98)	0.85 (0.62–1.16)	0.89 (0.64–1.25)
Q4	65	6837.3	0.54 (0.39–0.74)	0.68 (0.49–0.95)	0.79 (0.56–1.11)	0.84 (0.59–1.21)
*P* for trend			<0.001	0.03	0.18	0.31
PH assumption			0.54	0.63	0.69	0.62
**Stroke**						
Q1	19	4771.2	1	1	1	1
Q2	28	7396.5	0.96 (0.53–1.71)	0.78 (0.43–1.40)	1.05 (0.57–1.95)	0.98 (0.49–2.00)
Q3	15	7566.7	0.50 (0.25–0.98)	0.64 (0.32–1.25)	0.81 (0.41–1.63)	0.80 (0.38–1.69)
Q4	15	7050.7	0.53 (0.27–1.05)	0.83 (0.42–1.64)	1.15 (0.57–2.33)	1.29 (0.59–2.79)
*P* for trend			0.01	0.44	0.98	0.75
PH assumption			0.94	0.58	0.74	0.50
**CVD**						
Q1	99	4497.4	1	1	1	1
Q2	130	7003.8	0.85 (0.65–1.10)	0.73 (0.56–0.95)	0.93 (0.71–1.23)	0.99 (0.73–1.36)
Q3	96	7243.2	0.60 (0.45–0.79)	0.71 (0.53–0.94)	0.85 (0.63–1.13)	0.89 (0.65–1.21)
Q4	80	6777.4	0.53 (0.40–0.71)	0.70 (0.52–0.95)	0.86 (0.63–1.16)	0.92 (0.66–1.28)
*P* for trend			<0.001	0.02	0.24	0.47
PH assumption			0.54	0.43	0.48	0.38
**All-cause mortality**						
Q1	62	4815.5	1	1	1	1
Q2	105	7505.9	1.09 (0.79–1.49)	0.81 (0.59–1.11)	0.89 (0.63–1.24)	0.80 (0.54–1.19)
Q3	60	7630.5	0.61 (0.42–0.86)	0.77 (0.54–1.10)	0.89 (0.62–1.28)	0.81 (0.55–1.20)
Q4	34	7110.6	0.37 (0.24–0.56)	0.59 (0.39–0.90)	0.64 (0.41–0.98)	0.60 (0.38–0.96)
*P* for trend			<0.001	0.02	0.06	0.05
PH assumption			0.85	0.76	0.63	0.59
**CVD mortality**						
Q1	33	4815.5	1	1	1	1
Q2	41	7505.9	0.80 (0.51–1.27)	0.59 (0.37–0.94)	0.78 (0.47–1.28)	0.71 (0.39–1.30)
Q3	22	7630.5	0.42 (0.24–0.72)	0.54 (0.32–0.93)	0.70 (0.40–1.23)	0.61 (0.33–1.11)
Q4	9	7110.6	0.18 (0.09–0.38)	0.30 (0.14–0.63)	0.38 (0.18–0.82)	0.37 (0.17–0.83)
*P* for trend			<0.001	0.001	0.01	0.01
PH assumption			0.55	0.69	0.70	0.55

*Q, Quartile; HR (95% CI), Hazard ratio (95% confidence interval); MI, Myocardial Infraction; IHD, Ischemic heart disease (MI + Sudden cardiac death); CVD, Cardiovascular disease*.

a *Adjusted for age (year)*.

b *Additionally, adjusted for education (illiterate/primary school/more than primary school), residency (urban/rural), smoking status (never/past/current smoker), daily physical activity (METs–min/day), family history of cardiovascular disease (yes/no), diabetes mellitus (yes/no), hypertension (yes/no), hypercholesterolemia (yes/no), and aspirin use*.

c *Additionally, adjusted for body mass index (kg/m^2^), dietary factor including red meat, fish, fruit and vegetable, hydrogenated and non–hydrogenated vegetable oil, fast food, cereals, legumes, animal fats, sweets, soft drink, and beverages*.

## Discussion

We found that higher nut consumption was inversely related to MI, IHD, stroke, and major CVD events as well as all-cause and CVD mortality in Iranians after 13 years of follow-up in unadjusted model. After adjustment for age and sex, the lower major CVD events, and CVD mortality were still significant among the participants in the highest quartile of nut intake. After full adjustment for potential confounders, nut consumption had an inverse association with CVD mortality. Moreover, sensitivity analysis based on sex showed an inverse association between frequent consumption of nuts and risk of CVD mortality and all-cause death, only in men after full adjustment.

Our results are consistent with some other studies. In a meta-analysis of 20 prospective cohort studies, there was an inverse association between nut consumption and incident CVD, IHD, and all-cause mortality. Nevertheless, this study found no relationship between nut intake and stroke events (6). The results of the large prospective cohort of the Nurses' Health Study showed that the frequency of nut consumption had an inverse association with CVD. IHD and all-cause mortality but not with stroke mortality (17). The Physicians' Health Study showed an inverse association between nut consumption and CVD mortality but not with stroke (18). Other studies reported that nut consumption reduced the risk of CVD and stroke (8, 19, 20). In agreement to our finding, a recent cohort study of prospective urban rural epidemiological in 16 countries from five continents indicated that higher nut intake was associated with a lower risk of all-cause, CVD and non-CVD mortality but not MI, stroke, and major CVD risk in low-, middle-, and high-income countries (21). Similar to our results in men, some meta-analysis studies also showed that higher nut consumption lowered the risk of all-cause and/or CVD mortality (7, 8, 22). In line with our finding, the Iowa Women's Health Study in postmenopausal women showed no significant relationship between nut intake and risk of mortality from CHD but a weak association with all-cause death (23). Different results in men and women might be due to increased CVD and its risk factors in postmenopausal years (24). The possible mechanism may be because of losing the protective effect of estrogen on vasomotor tone regulation and its beneficial effects on CVD risk factors in postmenopausal women (25, 26). Pregnancy-related complications and more prevalence of systemic autoimmune diseases in women than men can accelerate the future risk of CVDs (27–29), and therefore, the effect of a healthy diet may be less in women than in men.

Nuts, either a particular type or in general, are beneficial to health and improve various cardiovascular risk factors (9, 10, 30–33). However, it is not exactly clear through which mechanisms nuts affect cardiovascular health (18). Nuts are nutrient-dense foods, and various studies have suggested different compounds, especially α-tocopherol, selenium, phenolic compounds, fiber, carotenoids, and phytosterols, for their health benefits (34).

The nuts' healthy fat profile can be protective against CVD (6). Fat accounts for 45.2–74.7% of the weight of different nuts. The fat content mostly consists of MUFA (mainly oleic acid) and PUFA, mainly alpha-linoleic acid (ALA), with <4–5% of saturated fatty acids. Compared with marine n-3 PUFAs, plan sources of ALA have less price and more global availability. The review of Fleming and Kris–Etherton indicated a beneficial effect of ALA for the primary and secondary prevention of CVD (35). It has been established that lowering the intake of saturated fatty acids accompanied by higher intakes of MUFA and PUFA reduces CVD risk and mortality (36). It has also been suggested that the favorable effects of nuts on cardiovascular health are due to their anti-inflammatory properties (37). Nevertheless, the studies have not reported consistent results, whereas some studies found no significant relation between nut consumption and inflammatory biomarkers (38, 39); others have found that nut intake improves inflammatory biomarkers (40, 41).

Nuts are also a good source of vegetable protein. Bernstein and colleagues reported that consumption of nuts instead of red meat had an inverse association with the risk of coronary disease after 26 years of follow-up in women (42). Nuts are important sources of amino acid L-arginine, a substrate for endothelium-derived nitric oxide synthesis in the blood vessel wall that acts as a vasodilator and regulates vascular tone (43). It has been shown that nut consumption improves endothelial function (38). Nut fiber content is also notable (4–11 g/100 g). Moreover, nuts are considered as one of the favorable sources of dietary magnesium (44). Guasch-Ferré and colleagues reported an inverse association between CVD and all-cause mortality risk and magnesium intake (19). However, in the Physicians' Health Study, after additional adjustment for magnesium and fiber intake, the inverse association between nut consumption and mortality did not change, leading the authors to suggest that the favorable effects of nuts on mortality may be exerted through other mechanisms (18).

A newer but less investigated hypothesis is the nut–microbiome interaction in the human gut. On the one hand, it has been suggested that some harmful microbial species in the gut microbiome may play a role in the pathogenesis of atherosclerosis through producing a pro-atherogenic agent, trimethylamine-N-oxide, from foods such as red meat and eggs (45). On the other hand, nuts are considered as potential prebiotics. Thus, nut consumption ameliorates the growth of health beneficial microbial species (46). Studies have shown favorable effects of nut consumption on the gut microbiome and also on cardiometabolic health as a result of nut–microbiome interaction (47). However, only a limited number of studies have investigated the nut–microbiome interaction, and for a better understanding, further studies are needed.

### Strengths and Limitations

Our study had some strength. To the best of our knowledge, it was the first study in the Middle East that investigated the relationship between nut consumption and CVD events. The prospective design of the present study strengthened the causal inference because the assessment of dietary intake of nuts was conducted before the incidence of the events and had three measurements. The study population was from three districts in Iran that offered us a heterogeneous socioeconomic status and a wide range of dietary intakes.

The limitations of this study should be considered. Although a validated FFQ was used for dietary data collection, we cannot exclude the existence of misclassification, similar to all epidemiologic studies. Moreover, our FFQ did not provide us with data on portion sizes. Therefore, we did not have any data about the total energy intake. Ideally, 24-h recalls should have also been acquired and the data combined for ideal nutritional intake ascertainment. Despite controlling for several confounders, we cannot ignore the effect of residual confounding. Finally, there were no data of other comorbidities responsible for well-known causes of death such as cancer and cerebral stroke and a comparison with the incidence of CVD.

## Conclusion

We concluded an inverse association between nut intake (including walnuts, almonds, pistachios, and hazelnuts) and lower risk of CVD events through an unadjusted model; however, it disappeared after full adjustment for potential confounders except for CVD mortality in men and total population and all-cause mortality only in men. To investigate the effect of individual types of nuts, such as walnuts, pistachios, almonds, etc., and different preparation methods (raw, roasted, and/or salted) on CVD risk and mortality, we need studies with longer follow-up periods.

## Data Availability Statement

The raw data supporting the conclusions of this article will be made available by the authors, without undue reservation.

## Ethics Statement

The studies involving human participants were reviewed and approved by Isfahan Cardiovascular Research Center, WHO Collaborating Center. The patients/participants provided their written informed consent to participate in this study.

## Author Contributions

NM, NS, MS, and HR designed research. FS and NG conducted research. NM and NG wrote the paper. JS-S provided major changes in the paper. RH analyzed data. All authors contributed to the article and approved the submitted version.

## Conflict of Interest

The authors declare that the research was conducted in the absence of any commercial or financial relationships that could be construed as a potential conflict of interest.
